# Targeted mutagenesis in rabbit using an engineered BhCas12b variant

**DOI:** 10.1093/jmcb/mjac076

**Published:** 2022-12-26

**Authors:** Yingqi Jia, Tian Wang, Ding Zhao, Zhiquan Liu, Tingting Sui, Siyu Chen, Jinze Li, Liangxue Lai, Zhanjun Li

**Affiliations:** Key Laboratory of Zoonosis Research, Ministry of Education, College of Animal Science, Jilin University, Changchun 130062, China; Key Laboratory of Zoonosis Research, Ministry of Education, College of Animal Science, Jilin University, Changchun 130062, China; Key Laboratory of Zoonosis Research, Ministry of Education, College of Animal Science, Jilin University, Changchun 130062, China; Key Laboratory of Zoonosis Research, Ministry of Education, College of Animal Science, Jilin University, Changchun 130062, China; Key Laboratory of Zoonosis Research, Ministry of Education, College of Animal Science, Jilin University, Changchun 130062, China; Key Laboratory of Zoonosis Research, Ministry of Education, College of Animal Science, Jilin University, Changchun 130062, China; Key Laboratory of Zoonosis Research, Ministry of Education, College of Animal Science, Jilin University, Changchun 130062, China; Key Laboratory of Zoonosis Research, Ministry of Education, College of Animal Science, Jilin University, Changchun 130062, China; CAS Key Laboratory of Regenerative Biology, Guangdong Provincial Key Laboratory of Stem Cell and Regenerative Medicine, South China Institute for Stem Cell Biology and Regenerative Medicine, Guangzhou Institutes of Biomedicine and Health, Chinese Academy of Sciences, Guangzhou 510530, China; Guangzhou Regenerative Medicine and Health Guang Dong Laboratory (GRMH-GDL), Guangzhou 510005, China; Institute for Stem Cell and Regeneration, Chinese Academy of Sciences, Beijing 100101, China; Key Laboratory of Zoonosis Research, Ministry of Education, College of Animal Science, Jilin University, Changchun 130062, China

**Dear Editor**,

The clustered regularly interspaced short palindromic repeat (CRISPR) and CRISPR-associated protein (CRISPR–Cas) system has exhibited powerful abilities to manipulate genomes of animals and plants (Knott and Doudna, 2018). Up to now, numerous Cas nucleases have been harnessed for genome editing in human cells, such as Cas9, Cas12a (also known as Cpf1), and Cas12b (also termed C2c1). Cas12b, a Class 2 type V-B CRISPR system, generates staggered double-strand breaks (DSBs) in the target DNA (Stella et al., 2017) and recognizes a distal 5′-T-rich protospacer adjacent motif (PAM) sequence (Shmakov et al., 2015), making it a complement to Cas9 (recognizing 5′-NGG-3′ PAM) in genome editing. Three Cas12b nucleases have been engineered for targeted genome editing in mammals or plants: BhCas12b v4 (Strecker et al., 2019), BvCas12b (Strecker et al., 2019), and AaCas12b (Teng et al., 2018). However, they have not been compared parallelly with each other. In this study, we compared the three Cas12b proteins for genome editing in mammalian cells.

It has been reported that BhCas12b v4, BvCas12b, and AaCas12b recognize 5′-DTTN-3′ (D = A, T, or G; N = A, T, G, or C), 5′-ATTN-3′, and 5′-TTN-3′ as PAM sequences, respectively (Supplementary Figure S1). To compare their editing efficiencies, eight target sites with qualified PAMs were selected in HEK293T cells to test the endogenous editing activity (Supplementary Table S3). Briefly, BhCas12b v4, BvCas12b, AaCas12b, and their corresponding sgRNAs were transiently transfected into HEK293T cells to compare the editing activities in parallel. The results of deep sequencing showed that BhCas12b v4 generated the highest efficient editing at seven target sites with average frequencies of indels ranging from 7.40% ± 2.56% to 55.72% ± 0.17% (Figure 1A). In comparison, BvCas12b displayed significantly lower editing efficiency with the average frequencies of indels ranging from 5.84% ± 0.08% to 33.02% ± 5.04% (Figure 1A) at all target sites. AaCas12b failed to yield high editing efficiency at all target sites (Figure 1A). We then compared the best-performed BhCas12b v4 with FnCpf1, a well-studied Cas12a nuclease recognizing 5′-TTN-3′ PAM, since they have similar PAM requirements. BhCas12b v4 showed significantly improved editing activity at four (50%) of eight tested target sites and comparable editing efficiency with FnCpf1 at the other four target sites (Supplementary Figure S2). Overall, these results demonstrated that the BhCas12b v4 system generated higher editing efficiency than BvCas12b, AaCas12b, and even FnCpf1, showing its potential as an efficacious gene editing tool.

**Figure 1:**
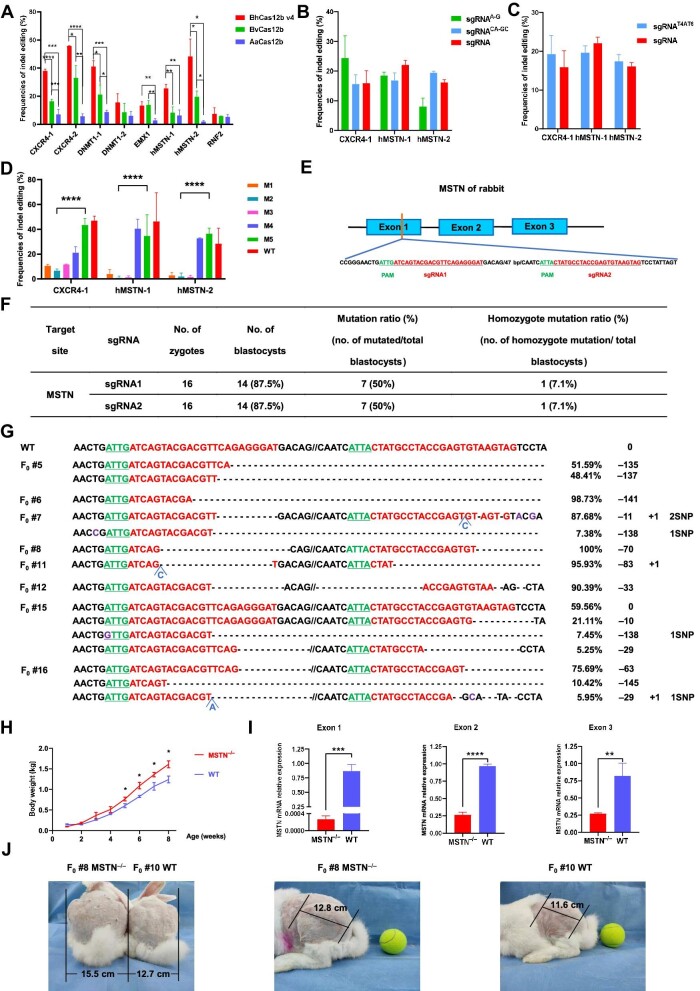
BhCas12b v4, BvCas12b, and AaCas12b induce efficient genome editing *in vivo*. (**A**) Comparison of indel editing efficiencies of BhCas12b v4, BvCas12b, and AaCas12b at eight genomic sites in HEK293T cells. The results are presented as mean value ± standard error of the mean of three independent experiments. (**B** and **C**) Editing frequencies of CXCR4-1, hMSTN-1, and hMSTN-2 were determined by using BhCas12b v4 and sgRNA^A-G^, sgRNA^CA-GC^, or sgRNA^T4AT6^ systems. (**D**) Indel editing efficiency of five different mutants at three sites in HEK293T cells. (**E**) Schematic diagram of sgRNA of the rabbit MSTN gene locus. Two sgRNA sequences are highlighted in red and the PAM sequences are presented in green. (**F**) BhCas12b v4-induced efficient and precise gene editing in rabbit embryos. (**G**) Mutation detection by deep sequencing in F_0_ pups. The WT sequence is shown at the top of the targeting sequence. The sgRNA sequences are shown in red, PAM sites are underlined and highlighted in green, SNP is shown in purple, and insertion is shown in blue. ‘–’, deletions; ‘+’, insertion. (**H**) Average body weight of MSTN^–/–^ and WT rabbits recorded weekly for 8 weeks (*n* = 3). (**I**) Expression of MSTN gene was determined by RT-qPCR. (**J**) Photos of MSTN^–/–^ (F_0_ #8) and WT (F_0_ #10) rabbits in F_0_. Body measurement was performed in rabbits at 12 weeks old.

Given that BhCas12b v4 is the most efficacious Cas12b, we tried to further improve its editing efficiency by introducing a C/G pair into sgRNA (Supplementary Figure S3A; Li et al., 2022) or adding T4AT6 sequences (Bin Moon et al., 2018) to the 3′-terminal of CRISPR RNA (crRNA) (Supplementary Figure S3B). These strategies were proved to improve editing efficiency of Cas12a or PE3 system by 2.31–2.77 folds, but they failed to significantly improve the editing efficiency of BhCas12b v4 (Figure 1B and C). A previous study has shown that optimization of sgRNA structure can improve the indel efficiency of CRISPR–Cas9 (Shojaei Baghini et al., 2021). We then applied this strategy to BhCas12b v4 to optimize its sgRNA. In BhCas12b v4 system, crRNA and trans-activating crRNA form a repetitive anti-repetitive duplex (Liu et al., 2017). We identified five potential modification sites (M1–M5) in total (Supplementary Figure S3C) and evaluated the indel efficiency of these five mutants among three target sites in HEK293T cells. The results showed that M4 and M5 did not significantly improve the efficiency compared with the original sgRNA, while the M1–M3 mutants did not produce indel at all (Figure 1D). The crystal lattice of AacCas12b–sgRNA binary complex indicates that part of its sgRNA structure is disordered (Liu et al., 2017). We speculated that BhCas12b v4 has a similar structure, and the regions of M4 and M5 mutations are located in the disordered region of sgRNA. These results lay the foundation for the design of smaller and simpler sgRNA and the exploration of Cas12b structure.

To evaluate the feasibility and efficiency of BhCas12b v4 *in vivo*, we tested it at two target sites with 5′-ATTN-3′ (PAM) in the myostatin (MSTN) gene in rabbit embryos (Figure 1E). A mixture of BhCas12b v4 mRNA and respective sgRNAs was microinjected into rabbit zygotes and cultured at the blastocyst stage. As shown in Figure 1F, 87.5% of injected embryos developed to the blastocyst stage. Then, 14 embryos were harvested and subjected to polymerase chain reaction (PCR) amplification. Sanger sequencing results showed that the mutation of MSTN was found in

seven tested blastocysts (50%) (Figure 1F; Supplementary Figure S4). These results showed that the dual sgRNA-directed BhCas12b v4 system is efficient for generation of mutations in the MSTN gene in rabbit embryos. MSTN, a member of the transforming growth factor-β superfamily, is important in integrating/mediating anabolic and catabolic responses, but the underlying mechanisms are only partially understood (Rodriguez et al., 2014). It is necessary to have a precise MSTN model to study these mechanisms. Cas12b has the characteristics of low off-target efficiency (Strecker et al., 2019). Therefore, we tried to generate a MSTN knock-out (KO) rabbit model using BhCas12b v4 system. A total of 132 zygotes microinjected with BhCas12b v4 mRNA and a sgRNA targeting MSTN were transferred into the oviducts of four surrogate rabbits. After a full-term gestation, three surrogate rabbits were pregnant to term and gave birth to 17 alive pups (Supplementary Figure S5 and Table S7). Out of 17 new born pups, 14 (82.35%) carried the target MSTN mutation. The target deep sequencing results showed that the indels ranged from 10 bp to 145 bp (Figure 1G; Supplementary Table S8).

In order to investigate the difference in muscle development between MSTN KO (MSTN^–/–^) and wild-type (WT) rabbits, their body weights were recorded weekly. As shown in Figure 1H, MSTN^–/–^ rabbits showed significant increase in body weight in comparison with WT rabbits. To further investigate whether the mutations induced by BhCas12b v4 disrupt the expression of MSTN gene in the rabbits, we performed real-time quantitative PCR (RT-qPCR) to analyse MSTN expression in the gluteus maximus of MSTN^–/–^ and WT rabbits using three sets of primers. As shown in Figure 1I, the expression of MSTN significantly reduced in MSTN^–/–^ rabbits compared with that of WT controls. A typical double-muscled phenotype was also observed in MSTN^–/–^ rabbits at the age of 12 weeks (Figure 1J). To detect the off-target effect in these rabbits, seven potential off-target sites were selected for PCR amplification and subjected to deep sequencing. The results showed that no editing event was detected in these off-target sites (Supplementary Figure S6). However, the off-target sites OT1 of MSTN-1 and OT2 of MSTN-2 are both located at chr13, which may cause large deletion due to the generation of two DSBs. To address this issue, we designed PCR primers flanking the two off-target sites. PCR results showed that there was indeed a large fragment deletion in one of the three rabbits tested (Supplementary Figure S7).

In summary, BhCas12b v4 was the most efficacious Cas12b nuclease as tested at endogenous genomic sites with T-rich PAM. It also induced higher editing efficiency than FnCpf1 at most target sites. By changing the structure of sgRNA, we explored the function of a part of sgRNA in Cas12b–sgRNA binary complex. To our knowledge, this is the first rabbit model with high gene mutation efficiency generated by Cas12b system. Given the small size and high efficiency of BhCas12b v4 (BhCas12b v4: 1108 amino acids; SpCas9: 1367 amino acids; and AsCas12a: 1307 amino acids), it is a promising tool for adeno-associated virus-mediated vector delivery *in vivo* and establishing animal models in the future.

*[Supplementary material is available at *Journal of Molecular Cell Biology* online. The authors thank Peiran Hu and Nannan Li at the Embryo Engineering Center for critical technical assistance. This study was financially supported by the National Key Research and Development Program of China Stem Cell and Translational Research (2022YFA1105404), the Program for Changjiang Scholars and Innovative Research Team in University (IRT_16R32), the Strategic Priority Research Program of the Chinese Academy of Sciences (XDA16030501 and XDA16030503), and Key Research & Development Program of Guangzhou Regenerative Medicine and Health Guangdong Laboratory (2018GZR110104004). Y.J., L.L., and Z.Li conceived and designed the experiments. Y.J., T.W., D.Z., and J.L. performed the experiments. Z.Liu and Y.J. analyzed the data. Y.J., S.C., and T.S. wrote the paper. All authors have read and approved the final manuscript.]*

## Supplementary Material

mjac076_Supplemental_File
