# Supplementary material for: Targeted mutagenesis in rabbit using an engineered BhCas12b variant
Source: J Mol Cell Biol. 2022 Dec 26;14(12):mjac076. doi: 10.1093/jmcb/mjac076 (PMC10129383; doi:10.1093/jmcb/mjac076)
Supplement: mjac076_Supplemental_File [file mjac076_supplemental_file.pdf]

Supplementary material

Targeted mutagenesis in rabbit using an engineered BhCas12b variant

Yingqi Jia[1,†], Tian Wang[1,†], Ding Zhao[1,†], Zhiquan Liu[1], Tingting Sui[1], Siyu Chen[1], Jinze Li[1], Liangxue Lai[1,2,3,4,*], and Zhanjun Li[1,*]

[1]Key Laboratory of Zoonosis Research, Ministry of Education, College of Animal Science, Jilin University, Changchun 130062, China

[2]CAS Key Laboratory of Regenerative Biology, Guangdong Provincial Key Laboratory of Stem Cell and Regenerative Medicine, South China Institute for Stem Cell Biology and Regenerative Medicine, Guangzhou Institutes of Biomedicine and Health, Chinese Academy of Sciences, Guangzhou 510530, China

[3]Guangzhou Regenerative Medicine and Health Guang Dong Laboratory (GRMH-GDL), Guangzhou 510005, China

[4]Institute for Stem Cell and Regeneration, Chinese Academy of Sciences, Beijing 100101, China

[†]These authors contributed equally to this work.

*Correspondence to: Liangxue Lai, E-mail: lai_liangxue@gibh.ac.cn; Zhanjun Li, E-mail: lizj_1998@jlu.edu.cn

Supplementary Methods

Ethics statement

New Zealand white rabbits were obtained from the Laboratory Animal Centre of Jilin University (Changchun, China). All animal studies were conducted according to experimental practices and standards approved by the Animal Welfare and Research Ethics Committee at Jilin University.

Plasmid construction

The BhCas12b v4, BvCas12b and AaCas12b plasmids were obtained from Addgene (#122446, #122445 and #121945). And their corresponding original sgRNA expression vectors also were obtained from Addgene (#122448, #122447 and #121958). M1–M5 plasmid site-directed mutagenesis was generated using the Fast Site-Directed Mutagenesis Kit (TIANGEN, Beijing) (Supplementary Table S1). The site-directed mutation primers are listed in Supplementary Table S2.

Cell culture and DNA transfection

The human embryonic kidney (HEK) 293T cell line was cultured in DMEM plus GlutaMax supplemented with 10% fetal bovine serum (HyClone), 1% MEM non-essential amino acids and 1% penicillin and streptomycin (Gibco) at 37 ℃ with 5% CO2. Cells were seeded into 6-well plates and transfected at 60%–75% confluency using Hieff Trans™ Liposomal Transfection Reagent (Yeasen, Shanghai, China) according to the manufacturer's protocol. After 48 h of transfection, genomic DNA was isolated and used for genotyping. The sequencing of sgRNA is listed in Supplementary Table S3 and the primers used for genotyping are listed in Supplementary Table S6.

mRNA and gRNA preparation

The BhCas12b v4 was linearized with BbsI, and mRNA was synthesized using the HiScribe™ T7 ARCA mRNA kit (NEB) *in vitro*. The mRNA was purified using the RNeasy Mini Kit (Qiagen) according to the manufacturer's instructions. A pair of annealed complementary oligonucleotides encoding was cloned into the BsmBI-digested gRNA expression vector (Supplementary Tables S4 and S5). The sgRNAs were *in vitro* transcription using the MAXIscript T7 kit (Ambion) and purified using miRNeasy Mini Kit (Qiagen) according to the manufacturer's protocol.

Microinjection of rabbit zygotes

The protocol used for the microinjection of pronuclear-stage embryos has been described in detail in our previously published study (Song et al., 2016). In brief, a mixture of gRNAs (40 ng/μl) and BhCas12b v4 (110 ng/μl) was injected into the cytoplasm of pronuclear stage embryos.

Single-embryo PCR amplification and rabbit genotyping

The single-embryo PCR amplification and rabbit genotyping has been described in our published study (Song et al., 2016). All the primers are listed in Supplementary Table S6.

Real-time quantitative PCR (RT-qPCR)

The total RNA was extracted from calf muscle with TRNzol-A+ reagent (TIANGEN, Beijing, China) according to the manufacturer's protocol. RNA was reverse transcribed to cDNA with DNase I (Fermentas) using the BioRT cDNA First Stand Synthesis Kit (Bioer Technology, Hangzhou, China). Primers used in this study are listed in Supplementary Table S9. RT-qPCR was performed using the BioEasy SYBR Green I Real Time PCR Kit (Bioer Technology, Hangzhou, China). The relative gene expression was determined by $2^{-\Delta\Delta CT}$ formula which was normalized to the Gapdh mRNA. All the data were repeated three times and expressed as mean ± SEM.

Off-target assay

The potential off-target sites for two target sites in MSTN$^{-/-}$ F_0 rabbits were predicted using online tool (http://www.rgenome.net/cas-offinder/). Off-targets sites and primers used to amplify target sequences were listed in Supplementary Tables S10 and S11.

Statistical analysis

The indel editing efficiencies of Cas12b-associated were determined by using deep sequencing. All data are expressed as mean ± SEM, with a minimum of three individual determinations in all experiments. The data were analysed using GraphPad prism software 8.0.2. A probability value smaller than 0.05 ($P < 0.05$) was considered statistically significant. *$P < 0.05$, **$P < 0.01$, ***$P < 0.001$, ****$P < 0.0001$.

Supplementary Reference

Song, Y., Yuan, L., Wang, Y., et al. (2016). Efficient dual sgRNA-directed large gene deletion in rabbit with CRISPR/Cas9 system. Cell Mol Life Sci *73*, 2959-2968.

Supplementary Figures

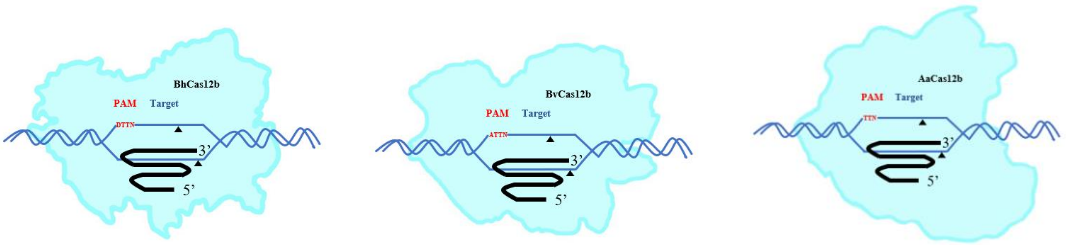

Supplementary Figure S1. Schematics of three activation systems tested, including BhCas12b v4, BvCas12b and AaCas12b with their corresponding sgRNA scaffolds.

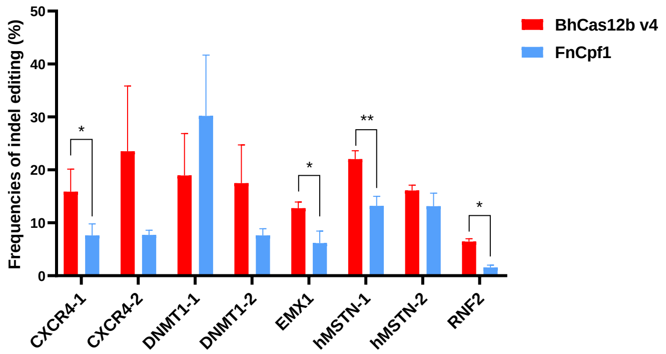

Supplementary Figure S2. Indels efficiency of BhCas12bv4 and FnCpf1.

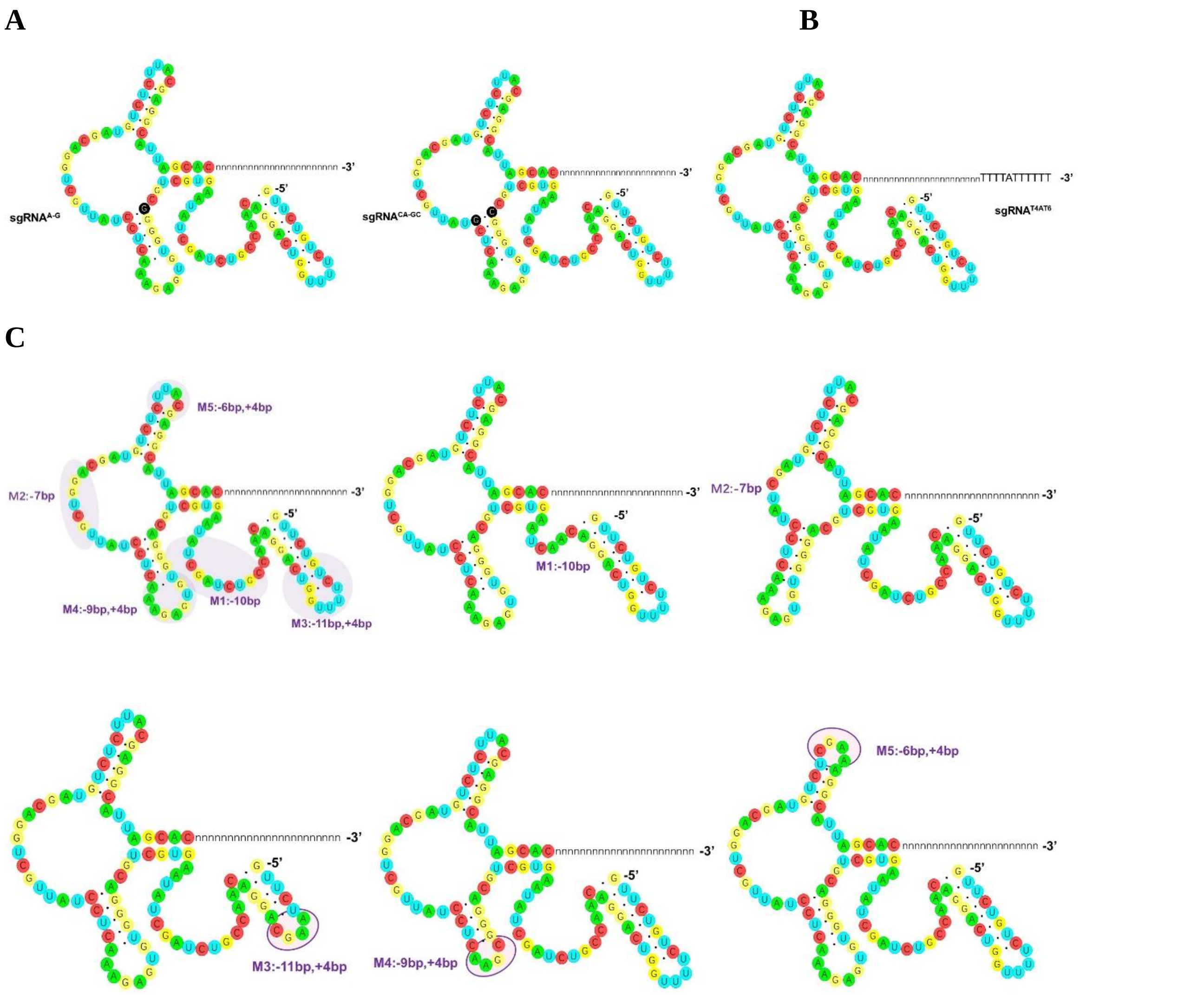

Supplementary Figure S3. Structure representation of the BhCas12b v4 gRNA. (**A**) CG base pairs were introduced into sgRNA. (**B**) T4AT6 sequence was added to the 3-terminal of sgRNA. (**C**) Five mutants for the gRNA project are showed.

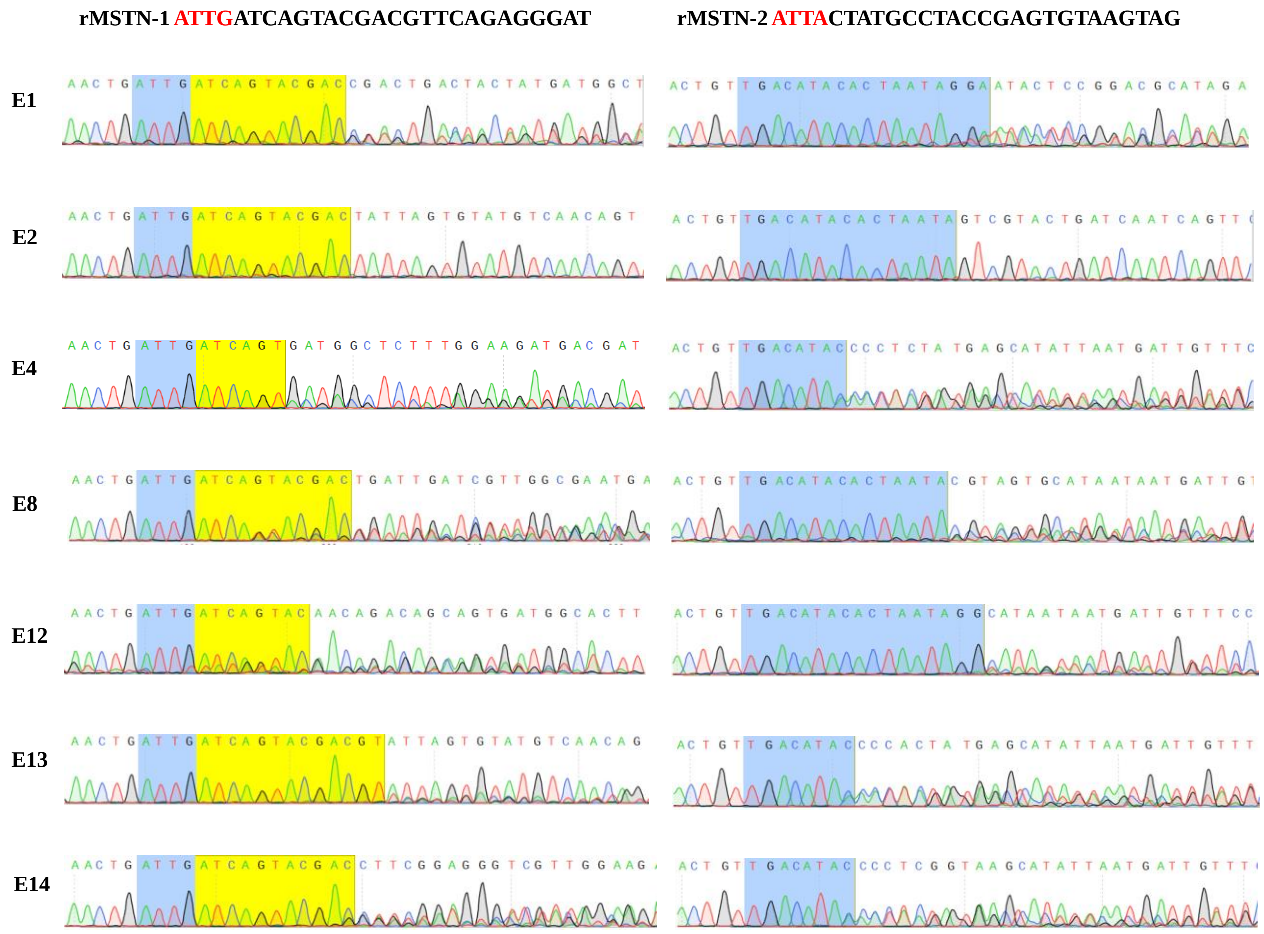

Supplementary Figure S4. Sanger sequencing chromatograms of BhCas12b v4 mediated indels in rabbit embryos.

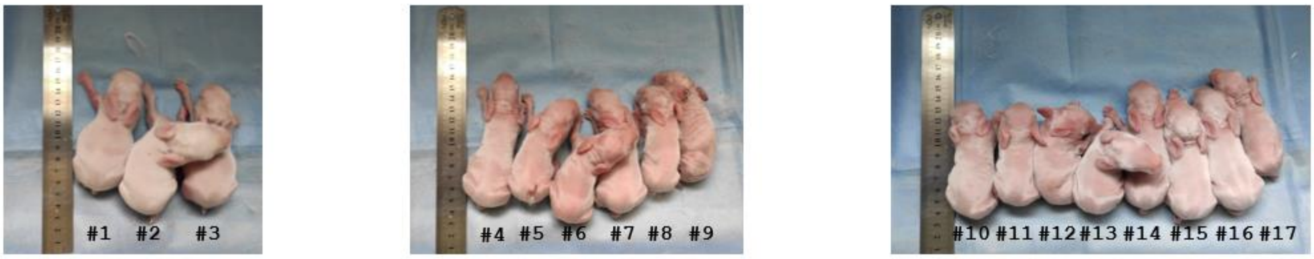

Supplementary Figure S5. Photographs of MSTN KO rabbits generated by BhCas12b v4 system.

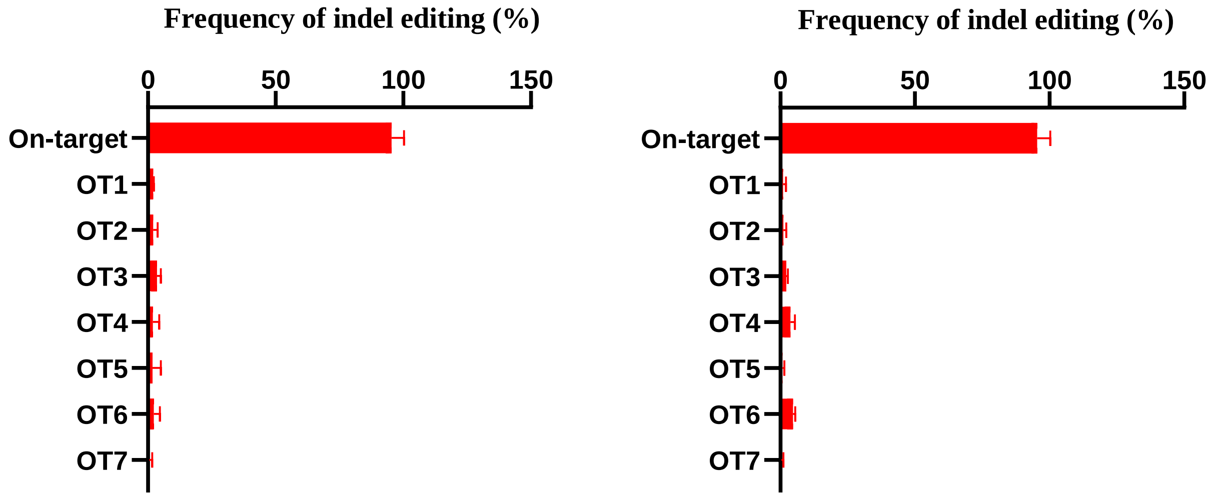

Supplementary Figure S6. On- and off-target frequency of indel editing in MSTN$^{-/-}$ rabbit (F0 #7) generated by the BhCas12b v4 system.

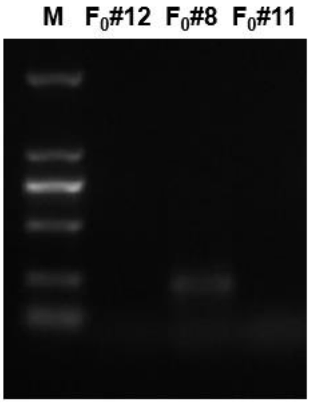

Supplementary Figure S7. Large fragment deletion in three rabbits tested by PCR. M (Marker2000).

Supplementary Tables

Supplementary Table S1. Sequences of BhCas12b v4 sgRNA variants. The position of deletion is indicated by red, and the position of insertion is indicated by green.

Description	Sequence
sgRNA-M1	GTTCTGTCTTCGAATTGGTCAGGACAACCGTCTAGCTATAAGTGCTGCAGG GTGTGAGAAACTCCTATTGCTGGACGATGTCTCTTACGAGGCATTAGCAC
sgRNA-M2	GTTCTGTCTTTTGGTCAGGACAACCGTCTAGCTATAAGTGCTGCAGGGTGT GAGAAACTCCTATTGCTGGACGATGTCTCTTACGAGGCATTAGCAC
sgRNA-M3	GTTCTGTCTTTTGGTCAGGACAACCGTCTAGCTATAAGTGCTGCAGGGTGT GACGAAGAAACTCCTATTGCTGGACGATGTCTCTTACGAGGCATTAGCAC
sgRNA-M4	GTTCTGTCTTTTGGTCAGGACAACCGTCTAGCTATAAGTGCTGCAGGGTGT GAGAAACTCCTATTGCTGGACGATGTCTCTTACGAGGCATTAGCAC
sgRNA-M5	GTTCTGTCTTTTGGTCAGGACAACCGTCTAGCTATAAGTGCTGCAGGGTGT GAGAAACTCCTATTGCTGGACGATGTCTCTTCGAAACAGGCATTAGCAC

Supplementary Table S2. The site-directed mutation primers used.

Description	Sequence
BhsgRNA-M1-F	TGTCTTTTGGTCAGGACAACTAAGTGCTGCAGGGTGTGAG
BhsgRNA-M1-R	TAGCTAGACGGTTGTCCTTTCGAGAACCGGTGTTTCGTCC
BhsgRNA-M2-F	AGGGTGTGAGAAACTCCTATCGATGTCTCTTACGAGGCAT
BhsgRNA-M2-R	ATGCCTCGTAAGAGACATCGATAGGAGTTTCTCACACCCT
BhsgRNA-M3-F	GGACGAAACACCGGTTCTCGAAAGGACAACCGTCTAGCTA
BhsgRNA-M3-R	CTCACACCCTGCAGCACTTAGTTGTCCTGACCAAAAGACA
BhsgRNA-M4-F	AGCTATAAGTGCTGCAGGGCGAACTCCTATTGCTGGACGA
BhsgRNA-M4-R	TCGTCCAGCAATAGGAGTTCGCCCTGCAGCACTTATAGCT
BhsgRNA-M5-F	ATTGCTGGACGATGTCTCGAAAGGCATTAGCACGGAGACG
BhsgRNA-M5-R	CGTCTCCGTGCTAATGCCTTTCGAGACATCGTCCAGCAAT

Supplementary Table S3. Sequences of sgRNA used in human HEK293T cells.

Description	Gene	Target	5' PAM	Sequence (5'–3')
BhCas12b v4 or BvCas12b	CXCR4	1	ATTC	TGGGCTTCAAGCAACTTGTAGTG
	CXCR4	2	ATTT	TGTAATTGGTTCTACCAAAGAAG
	DNMT1	1	TTTC	CCTCACTCCTGCTCGGTGAATTT
	DNMT1	2	ATTC	CTGGTGCCAGAAACAGGGGTGAC
	EMX1	1	ATTT	TTCATGGAGAAAATATTCAGAAT
	MSTN	1	ATTT	ACCTGTTTATGCTGATTGTTGCT
	MSTN	2	ATTA	AGATACAAATCCTCAGTAAACTT
	RNF2	1	TTTG	TAGTCATGGTGTTCTTCAACATA
AaCas12b	CXCR4	1	TTC	TGGGCTTCAAGCAACTTGTA
	CXCR4	2	TTT	TGTAATTGGTTCTACCAAAG
	DNMT1	1	TTC	CCTCACTCCTGCTCGGTGAA
	DNMT1	2	TTC	CTGGTGCCAGAAACAGGGGT
	EMX1	1	TTT	TTCATGGAGAAAATATTCAG
	MSTN	1	TTT	ACCTGTTTATGCTGATTGTT
	MSTN	2	TTA	AGATACAAATCCTCAGTAAA
	RNF2	1	TTG	TAGTCATGGTGTTCTTCAAC

Supplementary Table S4. Sequences of sgRNA used in rabbit embryos.

Gene	Target	5' PAM	Sequence (5'–3')
MSTN	1	ATTG	ATCAGTACGACGTTCAGAGGGAT
MSTN	2	ATTA	CTATGCCTACCGAGTGTAAGTAG

Supplementary Table S5. Primers used for mRNA *in vitro* transcription.

Description	Sequence
IVT-T7-F	TAATACGACTCACTATAGGGTTCTGTCTTTTGGTCAGGAC
IVT-MSTN-1-R	ATCCCTCTGAACGTCGTACTGAT
IVT-MSTN-2-R	CTACTTACACTCGGTAGGCATAG

Supplementary Table S6. Primers used for DNA genotyping in 293FT cells and rabbits.

Target species	Primer name	Primer sequence (5'–3')
Homo sapiens	CXCR4-1-F	CCACGCCTGCCTAAATACAA
	CXCR4-1-R	ATAAACACGAGGATGGCAAGAG
	CXCR4-2-F	GGCTAAGGGCACAAGAGAATTA
	CXCR4-2-R	CAGAGGAGTTAGCCAAGATGTG
	DNMT1-1-F	GACCCAATAAGTGGCAGAGT
	DNMT1-1-R	CAGGCCTTTGGTCAGGTT
	DNMT1-2-F	AAGGCCACAAACACCATGTA
	DNMT1-2-R	AGGAGGAGGAAGCTGCTAA
	EMX1-F	GCACAGGGAGAGGGTTTATG
	EMX1-R	GACTAGCAACACTGGCTCAA
	MSTN-1-F	AGCCACTTGGAATACAGTATAA
	MSTN-1-R	TTTGCTCACTGTTCTCATTTAG
	MSTN-2-F	GTTTATGCTGATTGTTGCTGGT
	MSTN-2-R	GTGGAGGAGCTTTGGGTAAA
	RNF2-F	ACAGGAGGCAATAACAGATGG
	RNF2-R	CACTTCTAAGGGCTGTGATGAT
Oryctolagus cuniculus	MSTN-1/2-F	TGGCCCAGTGGATCTAAATG
	MSTN-1/2-R	GAGACTGTCTTTCCTGCTTCTTA
Oryctolagus cuniculus deep sequencing	MSTN-1//2-F-Deep	CCTGGAAACAGCTCCTAACA
	MSTN-1/2-R-Deep	ACTAGAACAACAGTCAGCAGAA

Supplementary Table S7. Generation of genetically targeted MSTN-KO rabbits using BhCas12b v4 system.

Receptor	Cas12b/sgRNA mRNA(ng/μl)	Embryos injected	Embryos transferred (% microinjected)	Pregnancy	Pups obtained
1	25/100	37	34(92.5%)	YES	3
2	25/100	35	32(92.1%)	YES	6
3	25/100	39	33(85.7%)	YES	8
4	25/100	37	33(95.0%)	NO	0

Supplementary Table S8. The NSG genotyping of the MSTN model rabbits.

F_0 rabbit	Mutation type	The frequency of each mutation	Total mutation frequency
F_0 #5	-135	51.59%	100%
	-137	48.41%	
F_0 #6	-141	98.73%	100%
	-138	1.28%	
F_0 #7	-11 +1 2SNP	87.68%	91.24%
	-29	2.41%	
	-24	1.15%	
F_0 #8	-70	100%	100%
F_0 #11	-83 +1	95.93%	100%
	-69 +1 1SNP	2.32%	
	-83 +1 1SNP	1.76%	
F_0 #12	-33	90.39%	98.53%
	-137	4.59%	
	-33 2SNP	1.93%	
	-33 1SNP	1.62%	
F_0 #15	-10	21.11%	40.44%
	-138 1SNP	7.45%	
	-29	5.25%	
	-3	3.04%	
	-30	2.55%	
	-15	1.04%	
F_0 #16	-63	75.69%	97.63%
	-145	10.42%	
	-29 +1 1SNP	5.95%	
	-26	4.51%	
	-63 1SNP	1.06%	

Supplementary Table S9. RT-qPCR primers used for MSTN gene expression in rabbits.

Description	Forward	Reverse
Exon1	CTACGACGGAAACAATCATTAC	AAGAAGCAACATTTGGGTTT
Exon2	CCTGAGGCTCATCAAACCTATG	CACATCAATGCTCTGCCAAATAC
Exon3	GGTCATGATCTTGCTGTAACCT	TTGGTGTGTCCGTTACCTTG

Supplementary Table S10. The primers used for identifying potential off-target sites of MSTN$^{-/-}$ rabbit. The mismatched nucleotides are shown in lower case.

**Target site**	**Potential off-target sites**	**Mismatch number**	**Position**	**PCR primers**
MSTN-1	ATTCATCAaTACcACGTcCAGAGGGAa	4	chr13: +62634816	OT1-F: TCAAGGGAACACAGACGCA<br>OT1-R: GTCTCCTTTAGTCAAGTTACTGAAACC
	ATTTATCAGTAaGACagTaAGAGGGcT	5	chr12: -27670699	OT2-F: TGAAAGGTGAAGAGCAGCAT<br>OT2-R: ACTACAGCCTGCAAGTCTTTG
	GTTTATCAGgtgGAaGTTCtGAGGGAT	5	chr15: -98037975	OT3-F: GACAGTGTCAGAAATGGAAGAAAC<br>OT3-R: AAGCATTTAGCAGAGGTCCATAA
	ATTCtTgAGcAtGACtTTCAGAGGGAT	5	chr2: -10863197	OT4-F: TCAGGCTGCCCTTTGAAT<br>OT4-R: AGAACAGTGGGTAGTGTATATGTT
	ATTTATCAGTtCtAacTTCAGAGGcAT	5	chr2: -68253744	OT5-F: CTCTCTCTGTAACTCTGCCTTTAG<br>OT5-R: TGGATTAGTATAGACTTTGTATC
	ATTGATCAaTACaAgGcTCAGAGGGAa	5	chr3: +63079103	OT6-F: AGTCTTGGTAAACTGCAAGAAATG<br>OT6-R: GGTTTAACACAGTTCCTAGCTTTG
	GTTTATCtGTAtGACtgTCAGAGGGcT	5	chr3: +77150899	OT7-F: GAAGAGGGACCCAACCTTTAG<br>OT7-R: TGTAGAGTGGTATAATGTAACTTCCC
MSTN-2	TTTTCTATtCCTgaCtAGTGTAAcTAG	5	chr13: +78577025	OT1-F: CAGCTATGCTACCTCTACACTTC<br>OT1-R: ACCCAGAAAGTAGAACAAAGGA
	ATTGCaATGCCTAaacAGTGTgAGTAG	5	chr14: -158718621	OT2-F: TGTCATATCTGGTGGTGCAATA<br>OT2-R: ACTTAATCCTCTTCTGCTGGT
	GTTGCTtTGCCTcCaGAGTGTcAGgAG	5	chr18: +50156458	OT3-F: CAGCAGACTTGAGCTCCTTTA<br>OT3-R: GCTAGTTAGCTGGTATGAGTGG
	ATTTCTtTtCCTACCGAGTtTAgGTAt	5	chr2: +41155949	OT4-F: GGCCAAGAACCCTTAGGATAAT<br>OT4-R: AAACCAGGACCTAGAAAGTGAG
	GTTTCTATaCCaACaGAGTGTAAtaAG	5	chr20: +24962885	OT5-F: CCTCAGGGTACCTAAAGTTGATG<br>OT5-R: TCAAGGCCTGTCTGCATTC
	ATTCCTcTGCCTACaGAGTGgAgGaAG	5	chr3: -15298060	OT6-F: GTATCCTCACACTGGAGATCTTATC<br>OT6-R: TGGCGGCTTAACTTCCTATC
	ATTAaTtTttCTACtGAGTGTAAGTAG	5	chr4: +27112175	OT7-F: CATGTAAGCACAGCCCTCA<br>OT7-R: AAGGCATTAGAAGGCCTGAAA

Supplementary Table S11. The primers used to determine whether there is a large deletion between the off-target sites OT1 of MSTN-1 and OT2 of MSTN-2.

**Gene**	**Sequence (5'–3')**
MSTN-1-OT1-F	TCAAGGGAACACAGACGCA
MSTN-2-OT1-R	ACCCAGAAAGTAGAACAAAGGA
